# Four new species of *Cystolepiota* (Agaricaceae, Agaricales) from northeastern China

**DOI:** 10.3389/fmicb.2024.1358612

**Published:** 2024-04-04

**Authors:** Xian-Yan Zhou, Tolgor Bau

**Affiliations:** Key Laboratory of Edible Fungal Resources and Utilization (North), Ministry of Agriculture and Rural Affairs, Jilin Agricultural University, Changchun, China

**Keywords:** *Cystolepiota*, new species, phylogeny, taxonomy, northeastern China

## Abstract

*Cystolepiota* is a tiny lepiotaceous fungi. During our 3 years fieldwork, we found four new species of *Cystolepiota* from northeastern China. A phylogenetic study of a combined dataset of ITS+nrLSU+rpb2+tef1-α revealed that *Cystolepiota changbaishanensis* and *Cystolepiota hetieri* are sister clades; *Cystolepiota hongshiensis* belongs to *Cystolepiota seminuda* complex; *Cystolepiota luteosquamulosa* formed a clade not closely related with any other; *Cystolepiota nivalis* and *Cystolepiota* sp. (HMJAU68235) formed a sister clade. All new species are provided with descriptions, photos of the basidiomata, and colored illustrations of the microstructures. A key for the identification of *Cystolepiota* species from China is also presented.

## 1 Introduction

The humus layer of the forest harbors a myriad of tiny mushrooms that often go unnoticed, including *Cystolepiota* Singer. The genus *Cystolepiota* was erected by Singer in Singer and Digilio (1952) to accommodate small lepiotaceous fungi species with epithelioid squamules and inamyloid, non-dextrinoid basidiospores. Then Singer and Clémençon (1972) divided this genus into two sections: *C*. sect. *Pseudoamyloideae* Singer and Clémençon for species showing basidiospores with dextrinoid reactions in Melzer’s reagent (e.g., *Cystolepiota icterina* F. H. Møller ex Knudsen), and *C*. sect. *Cystolepiota* Singer with non-reactive basidiospores in Melzer’s reagent [e.g., *Cystolepiota fumosifolia* (Murrill) Vellinga]. In addition, Bon (1993) established a new genus, *Pulverolepiota* Bon, which includes species with the pileus covered by squamules formed by elongated and inflated cells, lacking clamp connections, and basidiospores slowly turning red brown in Melzer’s reagent [e.g., *C. petasiformis* (Murrill) Vellinga = *Pulverolepiota petasiformis* (Murrill) H. Qu, Damm and Z. W. Ge]. However, Vellinga treated this genus as a section of *Cystolepiota* (Vellinga and Huijser, 1998). Recently, Qu et al. (2023) found that *Pulverolepiota* formed a unique branch independent of the core members of *Cystolepiota*, and revived *Pulverolepiota* as a genus. Nevertheless, much controversy remains in the academic community regarding the boundaries of *Cystolepiota*.

Like *Cystolepiota*, the *Melanophyllum* Velen. (Velenovský, 1921) basidiomata pileus is also composed of loosely arranged spherical cells and hyphae. However, *Melanophyllum* has basidiomata with lamellae of a distinctive color, reddish or greenish, and it has ornamented basidiospores. Vellinga (2003) observed that *Melanophyllum*, although it has colored spores, belonged to the same evolutionary branch as *Cystolepiota*, instead of being related to *Agaricus* L., as proposed by Singer (1986). Qu et al. (2023) confirmed that *Melanophyllum* and *Cystolepiota* form a monophyletic group and that some species in the *C. seminuda* complex also have basidiospore ornamentation. Therefore, the relationship between these two genera is difficult to define.

In addition to the well-recognized *Cystolepiota* species, several species assumed to be *Lepiota* (Pers.) Gray have pileus surface squamules composed of chains of sphaerocyst cells. Knudsen (1978) transferred sect. *Echinatae* from *Lepiota* to *Cystolepiota* because of the presence of sphaerocysts on their pileus. However, he revised this view and later treated it as *Lepiota* sect. *Echinatae* (Knudsen, 1980). Bon (1991) included these species in *Echinoderma* (Locq. ex Bon) Bon. Then, phylogenetic studies (Vellinga, 2003; Hou and Ge, 2020) have shown that *Echinoderma* is polyphyletic, species with globose to ellipsoid basidiospores are members of *Lepiota* (e.g., *Lepiota omninoflava* Y. J. Hou and Z. W. Ge), whereas those with subcylindrical spores should be placed under *Echinoderma* [e.g., *Echinoderma asperum* (Pers.) Bon].

According to the Index Fungorum (http://www.indexfungorum.org/, accessed on December 19, 2023), more than 40 *Cystolepiota* species have been described. However, several species have rarely been found since publication (e.g., *C. constricta* Singer, the type species of *Cystolepiota*). Nine *Cystolepiota* species have been recorded in China: *C. adulterina* F. H. Møller ex Knudsen, *C. fumosifolia*, *C. hetieri* (Boud.) Singer, *C. pseudofumosifolia* M. L. Xu and R. L. Zhao, *C. pseudogranulosa* (Berk. and Broome) Pegler, *C. pseudoseminuda* Y. J. Hou, H. Qu and Z. W. Ge, *C. pyramidosquamulo*sa H. Qu and Z. W. Ge, *C. seminuda* (Lasch) Bon, and *C. squamulosa* (T. Bau and Yu Li) Zhu L. Yang (Mao et al., 1997; Bau and Li, 2004; Chou, 2010; Xu et al., 2016; Yang and Ge, 2017; Yang et al., 2019; Qu et al., 2023). Of these, *C. squamulosa* was a species previously discovered by our research team during a survey of species in northeastern China (Bau and Li, 2004). Before this survey, only three *Cystolepiota* species (*C. pseudoseminuda*, *C. seminuda*, and *C. squamulosa*) had been reported in northeastern China.

Through morphological and phylogenetic analyses, we identified four additional *Cystolepiota* species from northeastern China. Since the type species of *Cystolepiota*, *C. constricta*, has no available sequence in GenBank. The concept of *Cystolepiota* s.l. was used in this study to include all the species of *Cystolepiota* and *Melanophyllum* aforementioned.

## 2 Materials and methods

### 2.1 Morphological studies

Specimens were collected from northeastern China between June and September of 2021–2023. Photos of the basidiomata were taken during field collection and the macroscopic characteristics of the basidiomata were recorded, with color descriptions based on Kornerup and Wanscher (1963). Then specimens were dried using silica gel, and the specimens are currently stored in the Herbarium of Jilin Agricultural University (HMJAU). The colored illustrations are based on photos of the basidiomata in the field collection. Light microscopy (LM: Olympus CX33) was used to observe the microstructure, the samples were rehydrated in 5% KOH, and OPLENIC Pro v1.92 was utilized to measure the microstructure. Among them, in basidiospores, in the notation [*n*, *m*, *p*], *n* represents the number of basidiospores measured, of *m* basidiomata of *p* specimens, and *a* − *b* × *c* − *d* represents the minimum − maximum value of the length × width of the basidiospores, and *Q* = *a* − *b* represents the minimum − maximum value of the length/width of the basidiospores, *Q*_v_ = represents the average of the length/width of the basidiospores. Descriptive terminology follows terms proposed by Vellinga (1988) and Clémençon (2012).

To know accurately whether the basidiospore’s surface is ornamented or not, we treated the lamellae with gold spray after placing them on a carrier stage and observed the basidiospore surface under a scanning electron microscope (SEM: Zeiss MERLIN, EHT1-5Kv).

In addition, Congo red was used to stain the structures for better observation. To determine whether the basidiospores wall was amyloid or not, Melzer’s reagent was employed. Cresyl blue was used to detect the metachromatic reaction, while cotton blue revealed whether the basidiospores were cyanophilous.

### 2.2 Phylogenetic studies

DNA was extracted from dried specimens using the NuClean PlantGen DNA kit (CWBIO, Beijing, China). In PCR amplification, the primer pairs ITS1F/ITS (White et al., 1990; Gardes and Bruns, 1993), LR0R/LR5 (Vilgalys and Hester, 1990; Rehner and Samuels, 1994), 6F/RPB2-7.1R (Matheny, 2005), and EF1-983F/EF1-1567R (Rehner and Buckley, 2005) were used to amplify the sequences of four DNA regions, ITS, nrLSU, rpb2, and tef1-α, respectively. The PCR procedures followed Hou and Ge (2020): pre-denaturation at 94°C for 5 min, followed by 94°C for 50 s, annealing for 50 s, LSU and tef1-α at 50°C, ITS at 52°C, rpb2 at 55°C, extension at 72°C for 1 min, and 35 cycles. The PCR products were purified and sequenced by Sangon Biotech Co., Ltd. (Shanghai, China). The newly generated sequences were deposited in GenBank.^1^

The phylogenetic analysis included the available sequences of *Cystolepiota* and its closely related genera *Melanophyllum*, *Pulverolepiota*, *Echinoderma*, and *Lepiota*, according to Sánchez-García et al. (2020) study, *Coprinus comatus* (O. F. Müll.) Pers. and *Cop. sterquilinus* (Fr.) Fr. were selected as the outgroups. Finally, the analyzed matrix contains 179 ITS sequences, 54 nrLSU sequences, 30 rpb2 sequences, and 26 tef1-α sequences, which are listed in Table 1. Multiple sequences were compared using MAFFT v7.110 (Katoh et al., 2019), and the resulting alignments were manually checked and optimized in MEGA v7.0.26 (Kumar et al., 2016). Gap sites were removed with trimAl (Capella-Gutiérrez et al., 2009) using “-automated1” command. ModelFinder (Kalyaanamoorthy et al., 2017) was used to select the best-fit model using AIC criterion. A maximum-likelihood (ML) analysis was performed using raxmlGUI v2.0 with GTRGAMMAI as the model of evolution, and branch support was estimated over 1,000 bootstrap partitions (BP) with the rapid bootstrap option (Edler et al., 2021). Bayesian Inference phylogenies were inferred using MrBayes v3.2.6 (Ronquist et al., 2012) under partition model (2 parallel runs, 21,772,200 generations), in which the initial 25% of sampled data were discarded as burn-in. All phylogenetic graph results are exported for viewing in Figtree v1.4.3 (Rambaut, 2016).

**Table 1:** GenBank accession numbers, geographical origins, and voucher numbers of taxa used for the phylogenetic analyses.

Taxon	Country	Voucher	Genbank accession number			
			ITS	LSU	rpb2	tef1-α
*Coprinus comatus*	USA	iNat:65426592	MW989737	**-**	**-**	**-**
*Coprinus comatus*	Poland	CCM14	JQ901445	**-**	**-**	**-**
*Coprinus sterquilinus*	South Korea	18089	OM809735	**-**	**-**	**-**
*Cystolepiota bucknallii*	Italy	G Zecchin 490	JF907979	**-**	**-**	**-**
*Cystolepiota bucknallii*	Netherlands	ecv 1761	AY176458	**-**	**-**	**-**
***Cystolepiota changbaishanensis***	**China**	**HMJAU68222**	**OR947164**	**-**	**PP465921**	**PP465905**
***Cystolepiota changbaishanensis***	**China**	**HMJAU68223**	**OR947165**	**OR947176**	**-**	**PP465906**
***Cystolepiota changbaishanensis***	**China**	**HMJAU68224**	**OR947166**	**OR947177**	**-**	**PP465907**
***Cystolepiota changbaishanensis***	**China**	**HMJAU68225**	**OR947167**	**OR947178**	**-**	**PP465908**
***Cystolepiota changbaishanensis***	**China**	**HMJAU68221**	**OR947168**	**OR947179**	**-**	**-**
***Cystolepiota changbaishanensis***	**China**	**HMJAU68226**	**OR947169**	**-**	**-**	**-**
***Cystolepiota changbaishanensis***	**China**	**HMJAU68227**	**OR947170**	**-**	**-**	**-**
***Cystolepiota changbaishanensis***	**China**	**HMJAU68228**	**OR947171**	**-**	**-**	**-**
***Cystolepiota changbaishanensis***	**China**	**HMJAU68229**	**OR947172**	**-**	**-**	**-**
***Cystolepiota changbaishanensis***	**China**	**HMJAU68230**	**OR947173**	**-**	**-**	**-**
***Cystolepiota changbaishanensis***	**China**	**HMJAU68231**	**OR947174**	**-**	**-**	**-**
***Cystolepiota changbaishanensis***	**China**	**HMJAU68232**	**OR947175**	**-**	**-**	**-**
*Cystolepiota changbaishanensis*	China	KUN HKAS 78850	MN810142	MN810103	MN820978	MN820918
*Cystolepiota cystophora*	Costa Rica	DUKE-JJ87	U85332	U85297	**-**	**-**
*Cystolepiota fumosifolia*	USA	MICH18884	U85333	**-**	**-**	**-**
*Cystolepiota fumosifolia*	USA	ecv 3278	EF121817	**-**	**-**	**-**
*Cystolepiota hetieri*	Netherlands	ecv 2237	AY176459	**-**	**-**	**-**
*Cystolepiota hetieri*	Italy	782	JF907982	**-**	**-**	**-**
*Cystolepiota hetieri*	China	420526MF0093	MG694259	**-**	**-**	**-**
*Cystolepiota hetieri*	China	KUN HKAS 53554	MN810143	MN810102	MN820977	MN820917
*Cystolepiota hetieri*	China	KUN HKAS 84189	MN810139	MN810094	MN820976	MN820916
*Cystolepiota hetieri*	Canada	HRL0772	MH979434	**-**	**-**	**-**
*Cystolepiota hetieri*	Canada	HRL1277	MH979438	**-**	**-**	**-**
***Cystolepiota hongshiensis***	**China**	**HMJAU68202**	**OR947184**	**OR960530**	**PP465915**	**PP465901**
***Cystolepiota hongshiensis***	**China**	**HMJAU68203**	**OR947185**	**OR960531**	**PP465916**	**PP465903**
***Cystolepiota hongshiensis***	**China**	**HMJAU68204**	**OR947186**	**OR960532**	**PP465918**	**PP465904**
***Cystolepiota hongshiensis***	**China**	**HMJAU68205**	**OR947187**	**OR960533**	**PP465917**	**-**
***Cystolepiota hongshiensis***	**China**	**HMJAU68206**	**OR947188**	**-**	**-**	**-**
***Cystolepiota hongshiensis***	**China**	**HMJAU68207**	**OR947189**	**-**	**-**	**-**
***Cystolepiota hongshiensis***	**China**	**HMJAU68208**	**OR947190**	**-**	**-**	**-**
***Cystolepiota hongshiensis***	**China**	**HMJAU68209**	**OR947191**	**-**	**-**	**-**
***Cystolepiota hongshiensis***	**China**	**HMJAU68210**	**OR947192**	**-**	**-**	**-**
***Cystolepiota hongshiensis***	**China**	**HMJAU68211**	**OR947193**	**-**	**-**	**-**
***Cystolepiota hongshiensis***	**China**	**HMJAU68212**	**OR947194**	**-**	**-**	**-**
***Cystolepiota hongshiensis***	**China**	**HMJAU68213**	**OR947195**	**-**	**-**	**-**
***Cystolepiota hongshiensis***	**China**	**HMJAU68214**	**OR947196**	**-**	**-**	**-**
***Cystolepiota hongshiensis***	**China**	**HMJAU68215**	**OR947197**	**-**	**-**	**-**
***Cystolepiota hongshiensis***	**China**	**HMJAU68216**	**OR947198**	**-**	**-**	**-**
*Cystolepiota icterina*	Denmark	RE0909921	AY176460	**-**	**-**	**-**
*Cystolepiota luteohemisphaerica*	Ecuador	TL 11724	AM946477	AM946476	**-**	**-**
***Cystolepiota luteosquamulosa***	**China**	**HMJAU67711**	**OR233619**	**OR240263**	**PP465910**	**-**
***Cystolepiota luteosquamulosa***	**China**	**HMJAU67807**	**OR584135**	**OR584129**	**PP465911**	**-**
***Cystolepiota luteosquamulosa***	**China**	**HMJAU67808**	**OR584136**	**OR584130**	**PP465912**	**PP465900**
***Cystolepiota luteosquamulosa***	**China**	**HMJAU67809**	**OR584137**	**OR584131**	**PP465913**	**PP465902**
***Cystolepiota luteosquamulosa***	**China**	**HMJAU67810**	**OR584138**	**OR584132**	**PP465914**	**PP465899**
***Cystolepiota luteosquamulosa***	**China**	**HMJAU69060**	**OR936324**	**-**	**-**	**-**
*Cystolepiota luteosquamulosa*	USA	iNAT:147467243	OR168850	**-**	**-**	**-**
***Cystolepiota nivalis***	**China**	**HMJAU68217**	**OR947145**	**OR947180**	**-**	**PP465909**
***Cystolepiota nivalis***	**China**	**HMJAU68218**	**OR947146**	**OR947181**	**PP465922**	**-**
***Cystolepiota nivalis***	**China**	**HMJAU68219**	**OR947147**	**OR947182**	**PP465919**	**-**
***Cystolepiota nivalis***	**China**	**HMJAU68220**	**OR947148**	**OR947183**	**PP465920**	**-**
*Cystolepiota pseudofumosifolia*	China	KUN HKAS 104303	MN810150	MN810095	MN820973	MN820919
*Cystolepiota pseudofumosifolia*	China	KUN HKAS 105918	MN810152	MN810108	MN820974	MN820920
*Cystolepiota pseudofumosifolia*	China	KUN HKAS 84523	OP059090	**-**	**-**	**-**
*Cystolepiota pseudofumosifolia*	China	ZRL2011054	KF804000	**-**	**-**	**-**
*Cystolepiota pseudofumosifolia*	China	ZRL2012038	KF804001	**-**	**-**	**-**
*Cystolepiota pseudoseminuda*	China	KUN HKAS 73969	MN810144	MN810100	MN820979	MN820925
*Cystolepiota pseudoseminuda*	China	KUN HKAS 92275	MN810149	MN810101	MN820980	MN820926
***Cystolepiota pseudoseminuda***	**China**	**HMJAU68238**	**OR936165**	**-**	**-**	**-**
***Cystolepiota pseudoseminuda***	**China**	**HMJAU68239**	**OR936166**	**-**	**-**	**-**
***Cystolepiota pseudoseminuda***	**China**	**HMJAU68240**	**OR936167**	**-**	**-**	**-**
*Cystolepiota* aff. *pseudoseminuda*	Netherlands	4-X-1989, H.A.Huijser s.n.	AY176350	**-**	**-**	**-**
*Cystolepiota* aff. *pseudoseminuda*	Germany	GLM-F116532	OL898727	**-**	**-**	**-**
*Cystolepiota* aff. *pseudoseminuda*	USA	RA715-2	MK213366	**-**	**-**	**-**
*Cystolepiota* aff. *pseudoseminuda*	USA	iNAT:91477290	OM809356	**-**	**-**	**-**
*Cystolepiota pyramidalis*	Laos	HNL502500	MZ574554	MZ569511	**-**	**-**
*Cystolepiota pyramidalis*	Thailand	MFLU 12-1774	MZ574555	MZ569512	**-**	**-**
*Cystolepiota pyramidosquamulosa*	Italy	9247	JF907983	**-**	**-**	**-**
*Cystolepiota pyramidosquamulosa*	India	HATFD14-95	KU847887	**-**	**-**	**-**
*Cystolepiota pyramidosquamulosa*	China	KUN HKAS 53985	OP059088	OP059068	OP104341	OP141792
*Cystolepiota* cf. *rosea*	Italy	475	JF907978	**-**	**-**	**-**
*Cystolepiota* cf. *rosea*	Italy	781	JF907981	**-**	**-**	**-**
*Cystolepiota* cf. *rosea*	China	KUN HKAS 106737	OP059091	**-**	**-**	**-**
*Cystolepiota seminuda*	Germany	GLM F042189	OL898732	**-**	**-**	**-**
*Cystolepiota seminuda*	China	KUN HKAS 54211	OP059096	**-**	**-**	**-**
*Cystolepiota seminuda*	China	KUN HKAS 106016	OP059097	OP059071	OP104339	OP141795
*Cystolepiota seminuda*	China	KUN HKAS 106008	OP059098	**-**	**-**	**-**
*Cystolepiota seminuda*	China	KUN HKAS 84275	OP059093	OP059072	OP104340	OP141796
***Cystolepiota seminuda***	**China**	**HMJAU68241**	**OR936179**	**-**	**-**	**-**
***Cystolepiota seminuda***	**China**	**HMJAU68242**	**OR936180**	**-**	**-**	**-**
***Cystolepiota seminuda***	**China**	**HMJAU68243**	**OR936181**	**-**	**-**	**-**
***Cystolepiota seminuda***	**China**	**HMJAU68244**	**OR936182**	**-**	**-**	**-**
***Cystolepiota seminuda***	**China**	**HMJAU68245**	**OR936183**	**-**	**-**	**-**
***Cystolepiota seminuda***	**China**	**HMJAU68246**	**OR936184**	**-**	**-**	**-**
***Cystolepiota seminuda***	**China**	**HMJAU68247**	**OR936185**	**-**	**-**	**-**
***Cystolepiota seminuda***	**China**	**HMJAU68248**	**OR936186**	**-**	**-**	**-**
***Cystolepiota seminuda***	**China**	**HMJAU68249**	**OR936187**	**-**	**-**	**-**
***Cystolepiota seminuda***	**China**	**HMJAU68250**	**OR936188**	**-**	**-**	**-**
***Cystolepiota* aff. *seminuda* 1**	**China**	**HMJAU68191**	**OR936168**	**-**	**-**	**-**
***Cystolepiota* aff. *seminuda* 1**	**China**	**HMJAU68192**	**OR936169**	**-**	**-**	**-**
***Cystolepiota* aff. *seminuda* 1**	**China**	**HMJAU68193**	**OR936170**	**-**	**-**	**-**
***Cystolepiota* aff. *seminuda* 1**	**China**	**HMJAU68194**	**OR936171**	**-**	**-**	**-**
***Cystolepiota* aff. *seminuda* 1**	**China**	**HMJAU68195**	**OR936172**	**-**	**-**	**-**
***Cystolepiota* aff. *seminuda* 1**	**China**	**HMJAU68196**	**OR936173**	**OR960557**	**-**	**-**
***Cystolepiota* aff. *seminuda* 2**	**China**	**HMJAU68197**	**OR936174**	**OR960558**	**-**	**-**
***Cystolepiota* aff. *seminuda* 2**	**China**	**HMJAU68198**	**OR936175**	**OR960559**	**-**	**-**
***Cystolepiota* aff. *seminuda* 2**	**China**	**HMJAU68199**	**OR936176**	**OR960560**	**-**	**-**
***Cystolepiota* aff. *seminuda* 2**	**China**	**HMJAU68200**	**OR936177**	**-**	**-**	**-**
***Cystolepiota* aff. *seminuda* 2**	**China**	**HMJAU68201**	**OR936178**	**-**	**-**	**-**
*Cystolepiota* aff. *seminuda*	USA	iNAT:35546740	OM212829	**-**	**-**	**-**
*Cystolepiota* aff. *seminuda*	China	420526MF0264	MH142017	**-**	**-**	**-**
*Cystolepiota* sp.	China	KUN HKAS 105719	MN810151	MN810109	MN820975	MN820921
*Cystolepiota* sp.	USA	iNAT:92046005	OM972295	**-**	**-**	**-**
*Cystolepiota* sp.	Canada	S D Russell HRL1282	MH979429	**-**	**-**	**-**
*Cystolepiota* sp.	USA	iNAT:56783720	OM473834	**-**	**-**	**-**
*Cystolepiota* sp.	USA	iNAT:91679566	OM972500	**-**	**-**	**-**
*Cystolepiota* sp.	USA	iNAT:30997241	MZ293204	**-**	**-**	**-**
*Cystolepiota* sp.	USA	iNAT:91488451	OM972547	**-**	**-**	**-**
*Cystolepiota* sp.	China	KUN HKAS 56447	OP059087	**-**	**-**	**-**
*Cystolepiota* sp.	USA	S D Russell HRL2161	MH979462	**-**	**-**	**-**
*Cystolepiota* sp.	USA	iNAT:17334037	MK573889	**-**	**-**	**-**
*Cystolepiota* sp.	China	KUN HKAS 84333	OP059086	OP059066	OP104333	OP141790
*Cystolepiota* sp.	China	KUN HKAS 84177	OP059085	OP059067	OP104334	OP141791
*Cystolepiota* sp.	China	KUN HKAS 70454	MN810137	MN810091	MN820972	MN820915
*Cystolepiota* sp.	Germany	GLM-F107803	OL898733	**-**	**-**	**-**
*Cystolepiota* sp.	Germany	GLM-F107804	OL898734	**-**	**-**	**-**
*Cystolepiota* sp.	Germany	GLM-F042174	OL898731	**-**	**-**	**-**
*Cystolepiota* sp.	China	KUN HKAS 84188	OP059099	**-**	**-**	**-**
*Cystolepiota* sp.	USA	HRL2162	MH979463	**-**	**-**	**-**
*Cystolepiota* sp.	USA	iNAT:32078885	MW018878	**-**	**-**	**-**
*Cystolepiota* sp.	USA	JLF7486b	MT360313	**-**	**-**	**-**
*Cystolepiota* sp.	England	K(M):141927	MZ159361	**-**	**-**	**-**
*Cystolepiota* sp.	USA	iNAT:102198642	OQ871723	**-**	**-**	**-**
***Cystolepiota* sp.**	**China**	**HMJAU68237**	**OR936193**	**-**	**-**	**-**
***Cystolepiota* sp.**	**China**	**HMJAU68234**	**OR936194**	**-**	**-**	**-**
***Cystolepiota* sp.**	**China**	**HMJAU68235**	**OR936195**	**-**	**-**	**-**
***Cystolepiota* sp.**	**China**	**HMJAU68257**	**OR936196**	**-**	**-**	**-**
***Cystolepiota squamulosa***	**China**	**HMJAU68251**	**OR936197**	**-**	**-**	**-**
*Cystolepiota squamulosa*	China	110114MFBPC083	MW554270	**-**	**-**	**-**
*Cystolepiota squamulosa*	China	130822MFBPC309	MW554154	**-**	**-**	**-**
*Cystolepiota thailandica*	Thailand	MFLU 22-0017	MZ574556	MZ569513	OR122647	**-**
*Cystolepiota rhodella*	Laos	HNL501799	MZ574551	MZ569508	**-**	**-**
*Cystolepiota rhodella*	Thailand	MFLU 22-0019	MZ574552	MZ569509	MZ574090	**-**
*Cystolepiota rhodella*	Thailand	MFLU 09-0050	MZ574553	MZ569510	**-**	**-**
*Echinoderma asperum*	North Macedonia	KUN-HKAS106783	MN810133	MN810088	**-**	**-**
*Echinoderma flavidoasperum*	China	KUN-HKAS 87905	MN710147	MN810098	**-**	**-**
*Echinodema hystrix*	France	25-X-1998	AY176377	AY176378	**-**	**-**
*Lepiota alba*	China	KUN-HKAS 90371	MN810115	MN810075	**-**	**-**
*Lepiota castanea*	China	KUN-HKAS 84179	MN810119	MN810077	**-**	**-**
*Lepiota clypeolaria*	China	KUN-HKAS 87248	MN810123	MN810080	**-**	**-**
*lepiota echinaceum*	China	KUN-HKAS 105582	MN810155	MN810104	**-**	**-**
*Lepiota jacobi*	China	KUN-HKAS 48802	MN810138	GU199356	**-**	**-**
*Lepiota magnispora*	China	KUN-HKAS 61622	JN944089	JN940285	**-**	**-**
*Lepiota omninoflava*	China	KUN-HKAS 106734	MN810157	MN810092	**-**	**-**
***Lepiota omninoflava***	**China**	**HMJAU68258**	**OR936203**	**-**	**-**	**-**
***Lepiota subcastanea***	**China**	**HMJAU68259**	**OR936204**	**-**	**-**	**-**
***Lepiota subgracilis***	**China**	**HMJAU68260**	**OR936205**	**-**	**-**	**-**
*Melanophyllum eyrei*	South Korea	ASIS23988	KF953546	**-**	**-**	**-**
*Melanophyllum eyrei*	Sweden	TL6692	AY176493	**-**	**-**	**-**
*Melanophyllum haematospermum*	England	K(M):176342	MZ159454	**-**	**-**	**-**
*Melanophyllum haematospermum*	USA	HRL1115	MH979425	**-**	**-**	**-**
*Melanophyllum haematospermum*	South Korea	ASIS25547	KF953545	**-**	**-**	**-**
*Melanophyllum haematospermum*	Netherlands	ecv 2111	AY176494	**-**	**-**	**-**
*Melanophyllum haematospermum*	Canada	HRL1807	MH979452	**-**	**-**	**-**
*Melanophyllum haematospermum*	Italy	913	JF908498	**-**	**-**	**-**
*Melanophyllum haematospermum*	Netherlands	ecv2249	AF391038	**-**	**-**	**-**
*Melanophyllum haematospermum*	USA	ecv2517	AF391039	**-**	**-**	**-**
***Melanophyllum haematospermum***	**China**	**HMJAU68254**	**OR936198**	**-**	**-**	**-**
***Melanophyllum haematospermum***	**China**	**HMJAU68253**	**OR936199**	**-**	**-**	**-**
***Melanophyllum haematospermum***	**China**	**HMJAU68256**	**OR936200**	**-**	**-**	**-**
*Melanophyllum* sp.	South Korea	KA17-0334	MN294888	**-**	**-**	**-**
*Melanophyllum* sp.	USA	iNAT:91685346	OM809285	**-**	**-**	**-**
*Melanophyllum* sp.	USA	iNAT:58290738	MZ234091	**-**	**-**	**-**
*Melanophyllum* sp.	USA	FLAS: F-62773	MN945959	**-**	**-**	**-**
*Melanophyllum* sp.	USA	FLAS-F-61695	MH212052	**-**	**-**	**-**
***Melanophyllum* sp.**	**China**	**HMJAU68255**	**OR936206**	**-**	**-**	**-**
*Pulverolepiota oliveirae*	China	KUN HKAS 124759	OP059089	OP059069	OP104336	OP141793
*Pulverolepiota oliveirae*	Portugal	SMPM304	KY472789	**-**	**-**	**-**
*Pulverolepiota petasiformis*	Netherlands	ecv 1763	AF391037	**-**	**-**	**-**
*Pulverolepiota petasiformis*	UK	ecv 1872	AF391036	**-**	**-**	**-**
*Pulverolepiota* sp.	Hawaii	HAW: JKS140	MK412604	**-**	**-**	**-**
*Pulverolepiota* sp.	Hawaii	HAW: JKS143	MK412600	**-**	**-**	**-**
*Pulverolepiota* sp.	USA	S D Russell HRL1900	MH979456	**-**	**-**	**-**
***Pulverolepiota* sp.**	**China**	**HMJAU68236**	**OR947199**	**-**	**-**	**-**

New sequences generated for this study are in bold.

## 3 Results

### 3.1 Phylogenetic analyses

**Figure 1:**
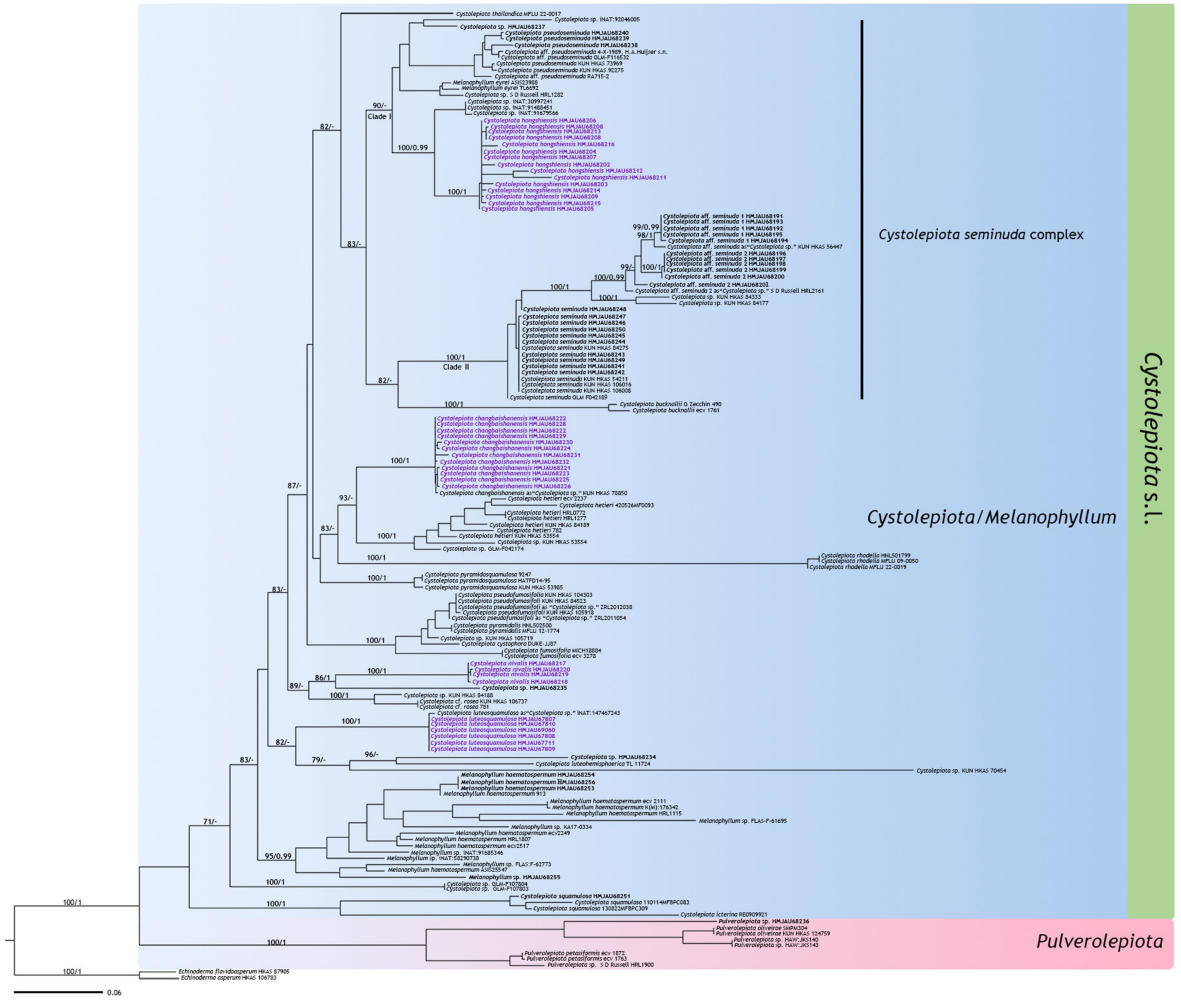
Maximum likelihood tree based on ITS sequences. New sequences generated for this study are in bold, new species sequences generated for this study are in purple bold. Bootstrap support (BS) values ≥70%, and Bayesian posterior probability (PP) values ≥0.95 are indicated on branches (BS/PP). *Echinoderma asperum* and *E. flavidoasperum* are used as outgroup.

**Figure 2:**
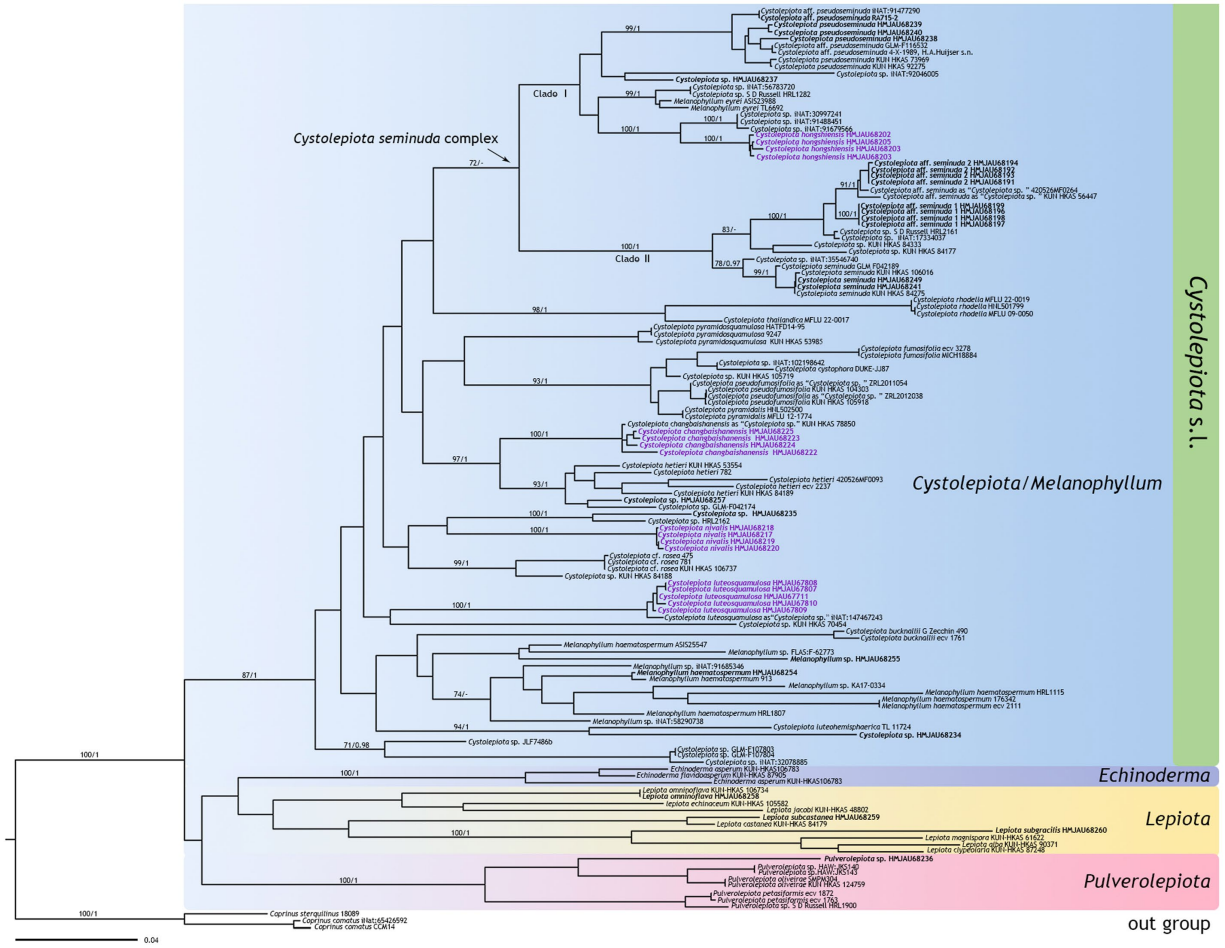
Phylogenetic tree of *Cystolepiota* obtained from the maximum likelihood analysis (ML) based on ITS, nrLSU, rpb2, and tefl-a sequence data. New sequences generated for this study are in bold, new species sequences generated for this study are in purple bold. Bootstrap support (BS) values ≥70%, and Bayesian posterior probability (PP) values ≥0.95 are indicated on branches (BS/PP). *Coprinus comatus* and *Cop. sterquilinus* are used as outgroup.

The ITS phylogenetic tree (Figure 1) included 154 sequences with 693 characters, and the multi-DNA regions phylogenetic tree (Figure 2) 133 sequences with 2,765 characters, including 133 ITS sequences, 54 nrLSU sequences, 30 rpb2 sequences, and 26 tef1-α sequences. BI and ML analysis resulted in a very similar topology, so the ML tree is provided in this study (Figures 1, 2). Bootstrap support (BS) values ≥70%, and Bayesian posterior probability (PP) values ≥0.95 are indicated on branches (BS/PP).

The four new species are distributed in different clades as follows: *Cystolepiota changbaishanensis* and *C. hetieri* are sister clades (Figure 1: BS/PP = 93/-; Figure 2: BS/PP = 97/1). *Cystolepiota hongshiensis* belongs to *C. seminuda* complex clade I, with a highly supported sister relationship with the clade formed by three specimens of *Cystolepiota* sp. (iNAT:30997241, iNAT:91488451, iNAT:91679566) (Figure 1: BS/PP = 100/0.99; Figure 2: BS/PP = 100/1). *Cystolepiota nivalis* and *Cystolepiota* sp. (HMJAU68235) also formed a sister clade (Figure 1: BS/PP = 86/1); and *Cystolepiota luteosquamulosa* formed a clade not closely related with any other (Figure 1: BS/PP = 82/-). In addition, *Cystolepiota* sp. (HMJAU68234, HMJAU68235, HMJAU68257), *Melanophyllum* sp. (HMJAU68255), and *Pulverolepiota* sp. (HMJAU68236) each form an independent clade on the phylogenetic trees (Figures 1, 2), which is not described here for the moment because only one specimen is available for observation.

### 3.2 Taxonomy

#### 3.2.1 *Cystolepiota changbaishanensis* T. Bau and X. Y. Zhou, sp. nov.

**Figure 3:**
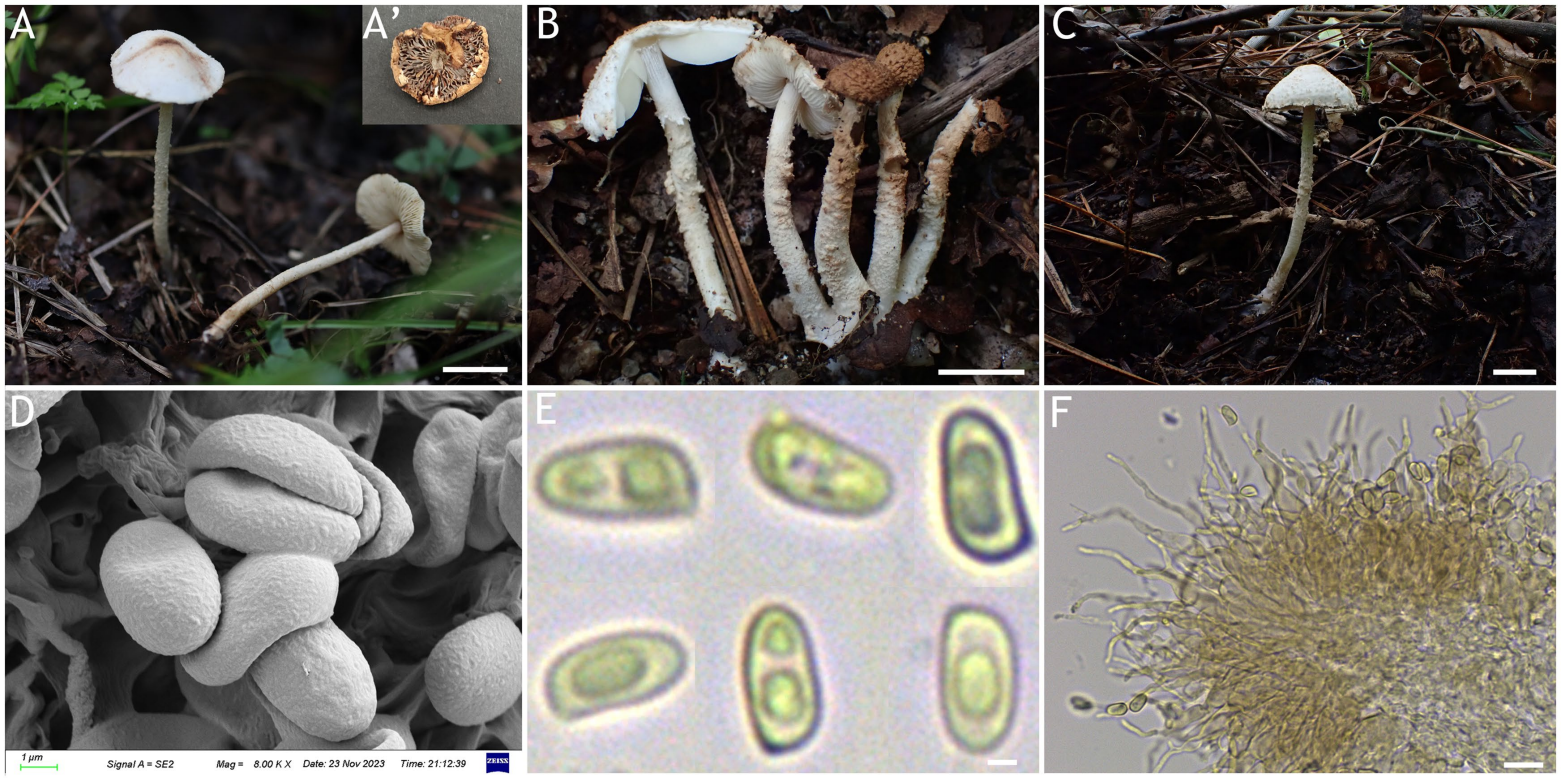
*Cystolepiota changbaishanensis*. **(A–C)** Basidiomata, **(A′)** dried specimen, **(D)** basidiospores under SEM, **(E)** basidiospores under LM, **(F)** cheilocystidia; **(A,D–F)** HMJAU68224 (holotype), **(B)** HMJAU68223, **(C)** HMJAU68222; bars: **A–C** =1 cm, **E** = 1 μm, **F** = 10 μm.

**Figure 4:**
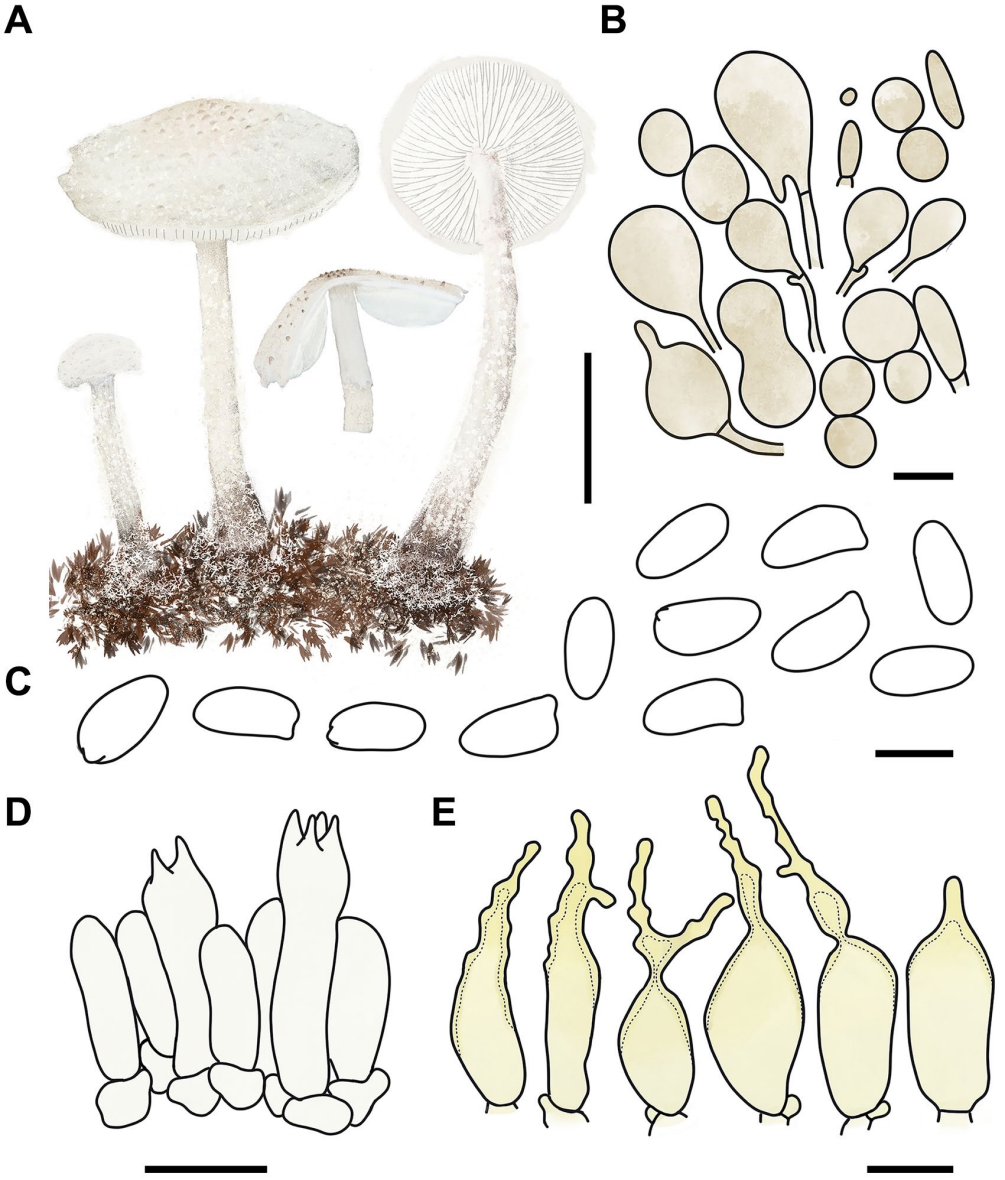
*Cystolepiota changbaishanensis*. **(A)** Basidiomata, **(B)** cells of the squamules, **(C)** basidiospores under LM, **(D)** basidia, **(E)** cheilocystidia; bars: **A** = 2 cm, **B** = 30 μm, **C** = 5 μm, **D,E** = 10 μm.

MycoBank number: MB 851389 (Figures 3, 4).

Diagnosis: The identifying features of *C. changbaishanensis* are that the pileus is dirty white to cream, with pulverulent, granulose or subpyramidal squamules, cream, greyish orange, light brown, brown; pileus and pileus context becoming greyish orange to brown after drying; lamellae white to cream, turn grayish orange to light brown when drying; basidiospores obscure small warts visible under SEM; and cheilocystidia lageniform to broadly lageniform.

Etymology: The species epithet “changbaishanensis” is derived from the name of the mountain where the material was collected.

Type: China, Jilin Province, Jiaohe City, Qianjin forest farm, July 23, 2022, coll. T. Bau and H. B. Song (HMJAU68224), Holotype!

Description: Basidiomata small. Pileus 0.8–2.2 cm, hemispherical when young, expanding to plano-convex or applanate, slightly subumbonate with age, dirty white to cream; with pulverulent, granulose or subpyramidal squamules, dirty white to cream, greyish orange (6B2–B8), light brown (7D5–D8), brown (7E5–E8); pileus context whitish, pileus and pileus context becoming greyish orange (6B5–B7) to brown (7E6–E8) after drying. Lamellae free, white to cream, crowded, up to 0.4 cm broad, with 1–3 tiers of lamellulae, turning grayish orange (6B2–B8) to light brown (6D2–D8) when drying. Stipe 3.2–6.2 × 0.1–0.5 cm, subcylindrical, occasionally downward thickened; white to cream on the upper portion, subsmooth, with granulose squamules from the annular area downwards, concolorous with pileus, fragile and fugacious. Annulus white, fugacious. Odor and taste not recorded (Figures 3A–C, 4A).

Basidiospores [150,5,5] 4.6–6.0 (−6.4) × 2.1–3.0 (−3.4) μm, *Q* = 1.63–2.54, *Q*_v_ = 2.08, long ellipsoid to cylindrical, hyaline, slightly thick-walled, smooth-walled under the LM, small warts visible under SEM, inamyloid, non-dextrinoid, metachromatic in cresyl blue, cyanophilous. Basidia13–20 × 4–7 μm, clavate, 4-spored, sometimes 2-spored, greyish yellow (4C4–C6). Lamellar trama regular, greyish yellow (4C3–C7). Cheilocystidia 32–56 × 6–12 μm, lageniform to broadly lageniform, greyish yellow (4C4–C7) to golden yellow (1B4–B8), with a long cylindrical-tortuous apex, slightly thick-walled. Pleurocystidia absent. Pileus and stipe covering an irregular epithelium composed of globose, subglobose, spheropedunculate, 10–21 μm in diam., usually 2–5 cells in a string, brownish orange (5C2–C5). Clamp connections present in all structures (Figures 3D–F, 4B–E).

Habitat: Solitary, scattered or clustered on dead leaves and soil of mixed coniferous forests.

Distribution: Found only in Jilin Province, northwestern China.

Additional specimens examined: China, Jilin Province, Helong City, Xianfeng National Forest Park, August 22, 2021, coll. T. Bau and X. Wang (HMJAU68221); Jiaohe City, Qianjin forest farm, July 23, 2022, coll. T. Bau, L. Y. Zhu, W. N. Hou and H. B. Song, (HMJAU68228, HMJAU68229, HMJAU68230); Dunhua City, State forest farm, July 27, 2022, coll. T. Bau and W. N. Hou (HMJAU68225); Baishan City, Jingyu National White Bear Reserve, July 29, 2022, coll. T. Bau and L. Y. Zhu (HMJAU682310); Tonghua City, Baijifeng National Forest Park, July 8, 2023, coll. T. Bau, Q. R. Liu, Z. Q. Cheng, M. Liu and J. L. Wei (HMJAU68226, HMJAU68227, HMJAU68232, HMJAU68222, HMJAU68223).

Notes: Macromorphologically, this species is similar to *C. fumosifolia*, *C. pyramidalis* and *C. pyramidosquamulosa*, because all of them present subpyramidal squamules on the pileus and stipe surface. But the lamellae of *Cystolepiota fumosifolia* usually have brown spots, and it has pleurocystidia (Vellinga, 2006). *Cystolepiota pyramidalis* has orange white to pale orange pileus, pale yellow lamellae, which turn brownish orange when touched or mature, and ellipsoid-ovoid basidiospores (Sysouphanthong et al., 2022). The lamellae of *Cystolepiota pyramidosquamulosa* are yellowish white, do not change color after drying, and do not have cystidia (Qu et al., 2023).

In the phylogenetic trees (Figures 1, 2), *Cystolepiota changbaishanensis* and *C. hetieri* are sister clades, but the lamellae of the latter’s basidiomata did not change color after drying and exhibited pleurocystidia.

#### 3.2.2 *Cystolepiota hongshiensis* T. Bau and X. Y. Zhou, sp. nov.

**Figure 5:**
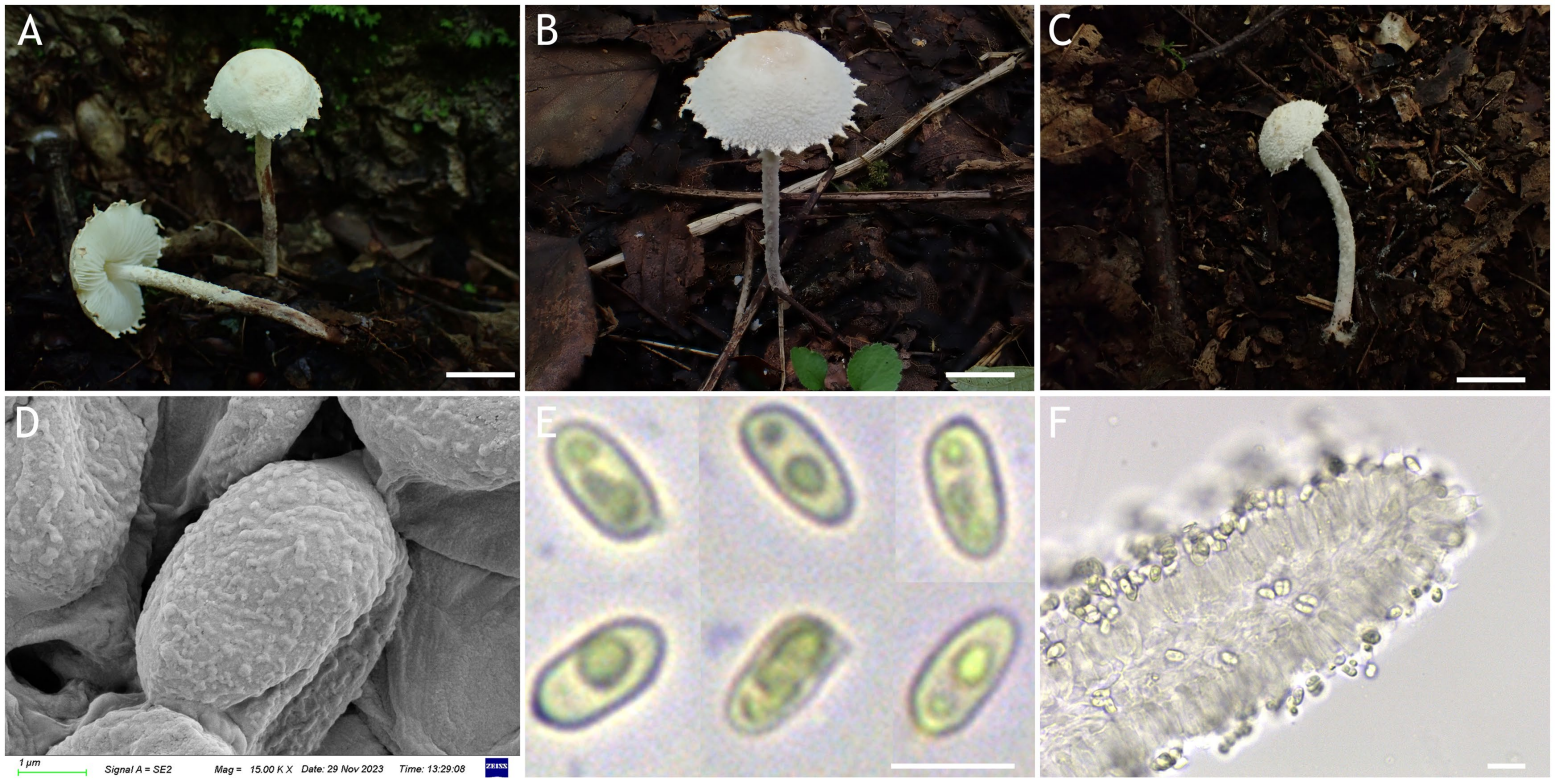
*Cystolepiota hongshiensis*. **(A–C)** Basidiomata, **(D)** basidiospores under SEM, **(E)** basidiospores under LM, **(F)** hyphae ends on the hymenium; **(A)** HMJAU68204 (holytype), **(B)** HMJAU68202, **(C)** HMJAU68207; bars: **A–C** = 1 cm, **E** = 5 μm, **F** = 10 μm.

**Figure 6:**
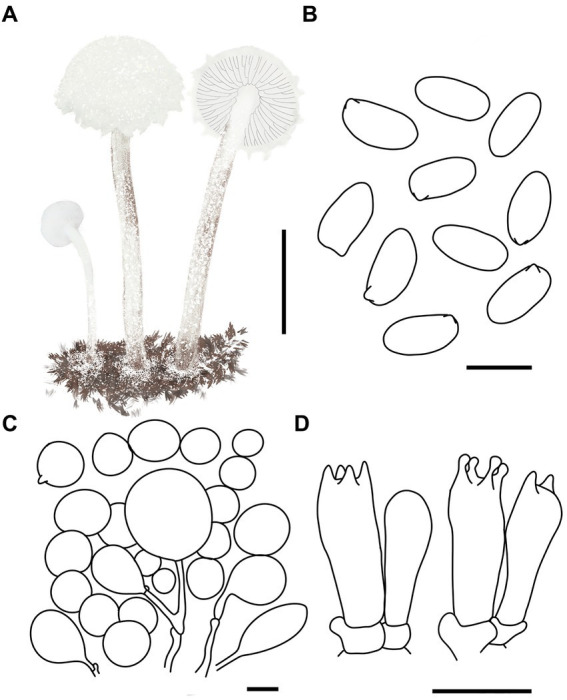
*Cystolepiota hongshiensis*. **(A)** Basidiomata, **(B)** basidiospores under LM, **(C)** cells of the squamules, **(D)** basidia; bars: **A** = 2 cm; **B** = 5 μm; **C** = 30 μm; **D** = 10 μm.

MycoBank number: MB 851390 (Figures 5, 6).

Diagnosis: *C. hongshiensis* is distinguished from other *Cystolepiota* species by its hemispherical to convex pileus, with granulose to warty squamules, white to cream, and rough basidiospores under SEM. Its ITS, LSU, rpb2, and tef1-α sequences are different from those of other species.

Etymology: The species epithet “hongshiensis” is derived from the name of the park where the material was collected.

Type: China, Jilin Province, Huadian City, Hongshi Township, Red Rock National Forest Park, August 27, 2023, coll. T. Bau and X. Wang (HMJAU68204), Holotype!

Description: Basidiomata small. Pileus 0.3–2.2 cm, hemispherical when young, hemispherical to convex with age, white to cream; with granulose to warty squamules, white to cream, yellowish white (4A2–A3), orange white (6A2–A3); occasionally pinkish orange (6A2–A3) on center, margin appendiculate with veil remnants when young, concolorous with pileus; pileus context white to cream. Lamellae free, white to cream, crowded, up to 0.3 cm broad, with 1–3 tiers of lamellulae. Stipe 2.1–5.3 × 0.1–0.2 cm, subcylindrical, slightly enlarged at base, surface white to cream on the upper portion, greyish orange (5B2–B3) to reddish brown (8E4–E8) at base, with age gradually turning to reddish brown (8E4–E8) towards the middle and lower portion, with pulverulent to granulose squamules, concolorous with pileus, fugacious; context reddish brown (8E4–E8) at stipe base. Annulus white, fugacious. Odor and taste not recorded (Figures 5A–C, 6A).

Basidiospores [120,4,4] (−3.7) 4.4–5.9 (−6.1) × 2.0–3.5 μm, *Q* = 1.53–2.30, *Q*_v_ = 1.89, long ellipsoid, hyaline, thin-walled, smooth-walled under the LM, distinct warts visible under SEM, inamyloid, non-dextrinoid, metachromatic in cresyl blue, cyanophilous. Basidia 15–22 × 5–7 μm, clavate, 4(2)-spored. Lamellar trama regular. Pleurocystidia and cheilocystidia absent. Pileus and stipe covering an irregular epithelium composed of globose to subglobose elements, 10–43 μm in diam., usually 2–5 cells forming loosely arranged chains, hyphae 1–4 μm in diam., slightly thick-walled, Clamp connections present in all structures (Figures 5D–F, 6B–D).

Habitat: Solitary to scattered on dead branches and rotten leaves of mixed forest.

Distribution: Found only in Jilin Province, northwestern China.

Additional specimens examined: China, Jilin Province, Jiaohe City, Qianjin forest farm, August 25, 2022, coll. T. Bau and H. Cheng (HMJAU68205); Jiaohe City, Hongyegu, July 31, 2023, coll. T. Bau and S. Y. Li (HMJAU68216); Huadian City, Zhaodaji Mountain National Forest Park, August 21, 2023, coll. T. Bau and X. Wang (HMJAU68207); Huadian City, Red Rock National Forest Park, August 27, 2023, coll. T. Bau, M. L. and X. Y. Zhou (HMJAU68203, HMJAU68206); August 28, 2023, coll. T. Bau, H. Cheng and X. Y. Zhou (HMJAU68202, HMJAU68212, HMJAU68215).

Notes: Macromorphologically, *Cystolepiota hongshiensis* and *C. pseudoseminuda*, with similar pileus surface squamules. But the latter pileus is plano-convex or applanate slightly umbonate, basidiospores (−3) 3.5–4.5 (−5) × 2–3 (−3.5) μm, *Q* = (−1.21) 1.24–1.85 (−2.20), *Q*_m_ = 1.55 ± 0.19, ovoid to ellipsoid, the basidiospores of *C. hongshiensis* are more elongated than those of *C. pseudoseminuda* (Qu et al., 2023). In addition, there are 65 (out of 706) nucleotide differences between the ITS sequences of the holotype of *C. hongshiensis* and that of the holotype of *C. pseudoseminuda*.

#### 3.2.3 *Cystolepiota luteosquamulosa* T. Bau and X. Y. Zhou, sp. nov.

**Figure 7:**
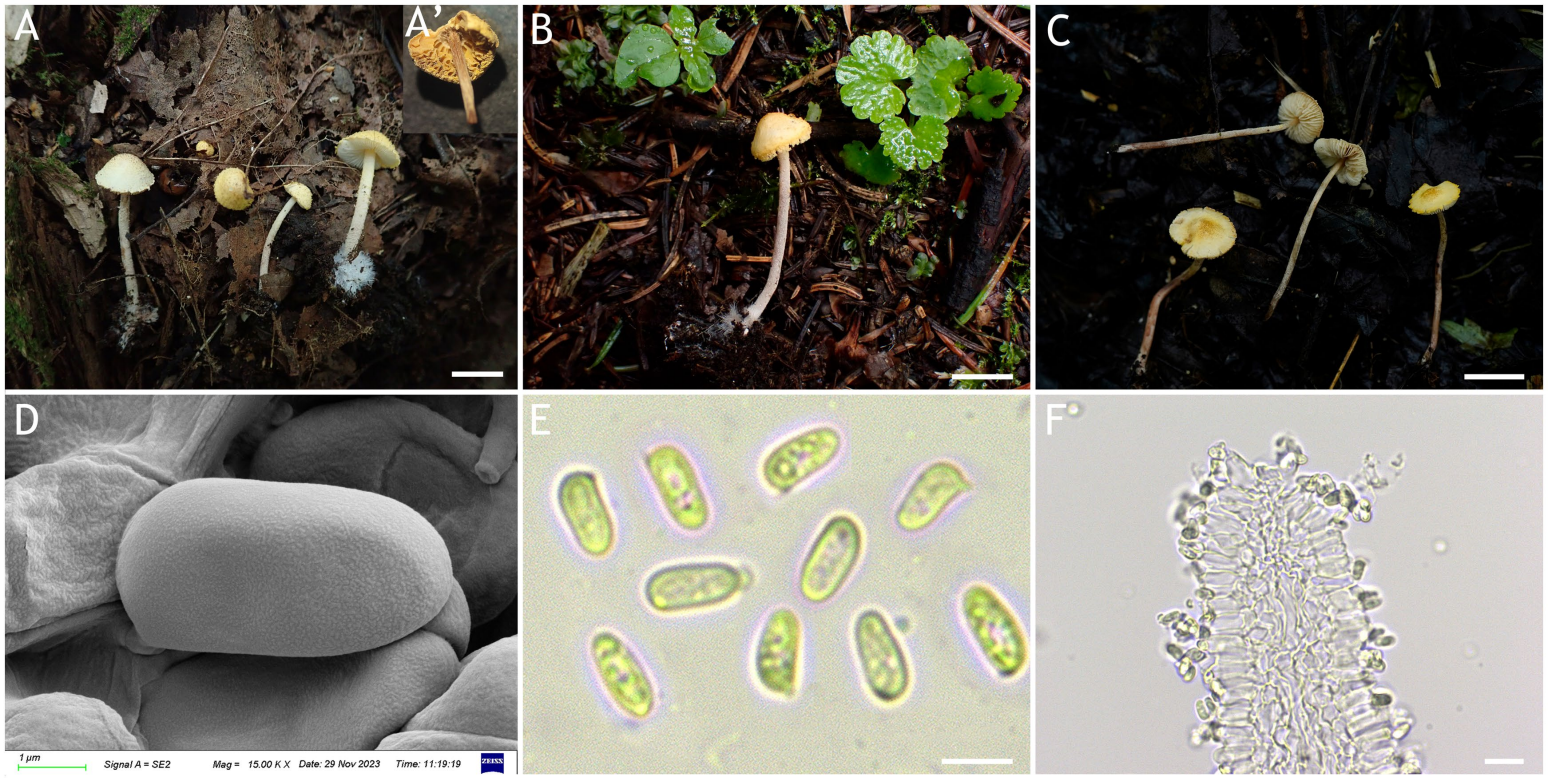
*Cystolepiota luteosquamulosa*. **(A–C)** Basidiomata, **(A′)** dried specimen, **(D)** basidiospores under SEM, **(E)** basidiospores under LM, **(F)** hyphae ends on the hymenium; **(A,D–F)** HMJAU67711 (holotype), **(B)** HMJAU67809, **(C)** HMJAU67810; bars: **A–C** = 1 cm.

**Figure 8:**
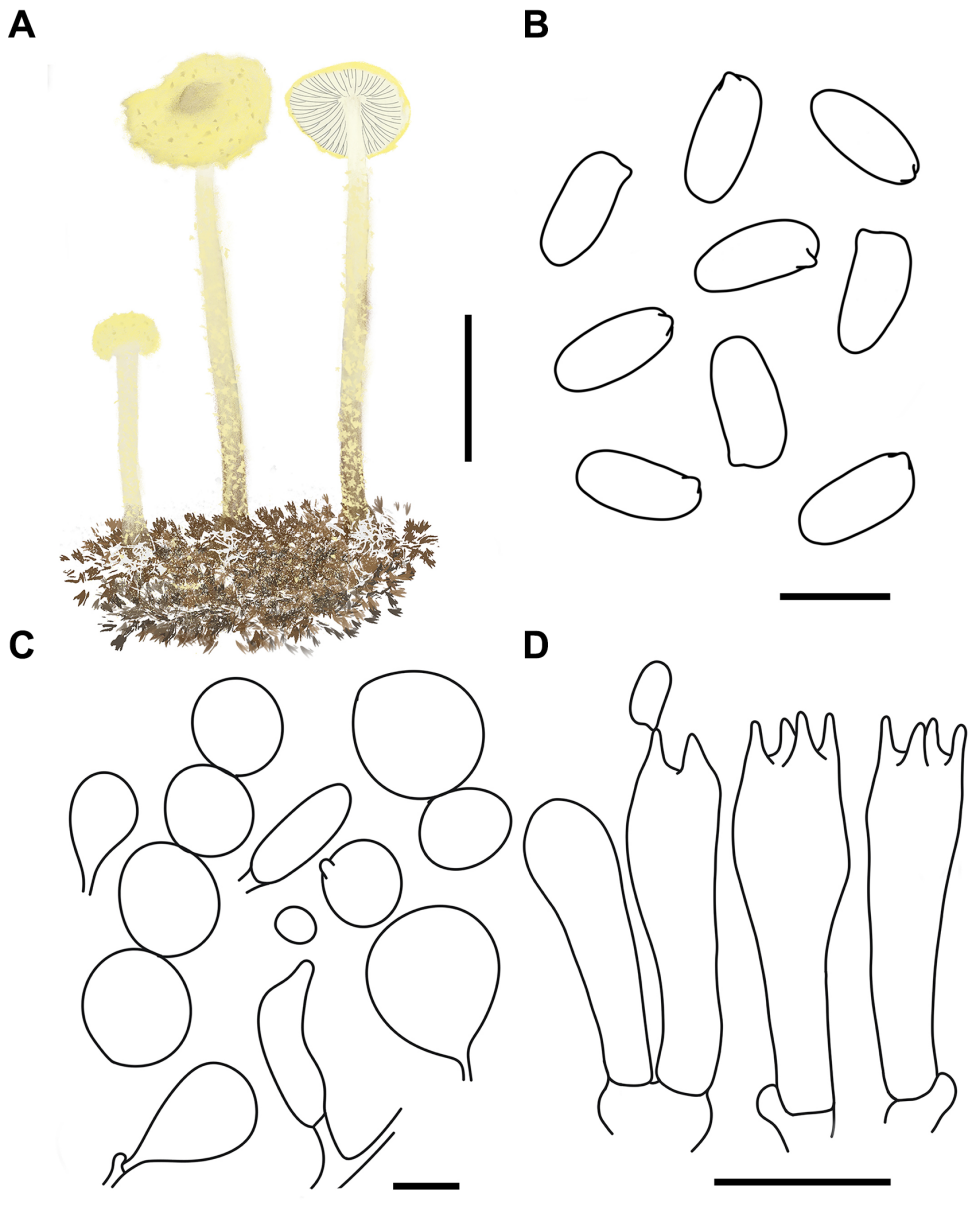
*Cystolepiota luteosquamulosa*. **(A)** Basidiomata, **(B)** basidiospores under LM, **(C)** cells of the squamules, **(D)** basidia; bars: **A** = 2 cm; **B** = 5 μm; **C** = 30 μm; **D** = 10 μm.

MycoBank number: MB 849380 (Figures 7, 8).

Diagnosis: *C. luteosquamulosa* is distinguished from other *Cystolepiota* species by its light yellow to greyish yellow pileus, with greyish yellow to dark yellow warty to subpyramidal squamules, light reddish brown stipe with white to light yellow floccose squamules, and pleurocystidia and cheilocystidia absent.

Etymology: “luteo-” means yellow, and “luteosquamulosa” refers to the yellow squamules on the pileus.

Type: China, Jilin province, Jiaohe City, Hongyegu, September 6, 2021, coll. T. Bau and X. Wang (HMJAU67711), Holotype!

Description: Basidiomata small. Pileus 0.8–1.4 cm, hemispherical to obtusely conical when young, expanding to plano-convex or applanate with a slightly umbonate center with age, light yellow (1A4–A8) to greyish yellow (2B5–B8), with greyish yellow (2C7–C8) to dark yellow (3C5–C8) warty to subpyramidal squamules; margin appendiculate with veil remnants when young, and then finely appendiculate, concolorous with pileus; context white, thin. Lamellae free, white to cream, crowded, 0.1–0.3 cm wide, with 1–3 tiers of lamellulae, drying brownish orange (5C2–C6). Stipe 2.5–5.6 × 0.1–0.2 cm, subcylindrical, light reddish brown (7E5–E8), with some floccose squamules, white to light yellow (1A2–A8), with conspicuous white mycelia on the base. Annulus not visible. Odor and taste not recorded (Figures 7A–C, 8A).

Basidiospores [120,4,4] 4.9–6.3 (−6.6) × (−2.0) 2.4–3.0 (−3.3) μm, *Q* = 1.70–2.63, *Q*_v_ = 2.10, long ellipsoid to cylindrical, hyaline, smooth-walled under the LM, finely punctate under SEM, inamyloid, non-dextrinoid, metachromatic in cresyl blue, cyanophilous. Basidia 15–23 × 4–7 μm, clavate, 4-spored, sometimes 2-spored. Lamellar trama regular. Pleurocystidia and cheilocystidia absent. Squamules composed of loosely-arranged globose, subglobose, ovoid, 13–62 μm in diam., rarely gourd-shaped or fusiform, 17–42 × 7–16 μm, sometimes 2–4 cells are connected in a string, smooth-walled, slightly thick-walled, hyaline, or orange white (5A2–A3). Clamp connections present in all structures (Figures 7D–F, 8B–D).

Habitat: Solitary or scattered on dead leaves or soil of mixed forest.

Distribution: Northeastern China.

Additional specimens examined: China, Jilin Province, Jiaohe City, Qianjin forest farm, July 24, 2022, coll. T. Bau and L. Y. Zhu (HMJAU67810); Dunhua City, State Forest farm, July 27, 2022, coll. T. Bau, W. N. Hou and F. Guo (HMJAU67808, HMJAU69060); Huadian City, Red Rock National Forest Park, August 28, 2023, coll. T. Bau and H. Cheng (HMJAU67807). Heilongjiang Province, Yichun City, Xing’an National Forest Park, July 25, 2023, coll. T. Bau and W. N. Hou (HMJAU67809).

Notes: Macromorphologically, both *C. luteosquamulosa* and *C. luteohemisphaerica* have yellow pileus. But in the latter, the pileus is radially veined and micaceous-mealy, with broadly elliptical to elliptical basidiospores (Saar and Laessoe, 2008). *C. icterina* also has a yellow pileus, but it is easy to distinguish from *C. lutesquamulosa* by its pileus surface with finely floccose-farinose squamules, by its smaller (3.5–4.5 × 2.5 μm) and dextrinoid basidiospores and by the presence of cheilocystidia (Knudsen, 1978).

#### 3.2.4 *Cystolepiota nivalis* T. Bau and X. Y. Zhou, sp. nov.

**Figure 9:**
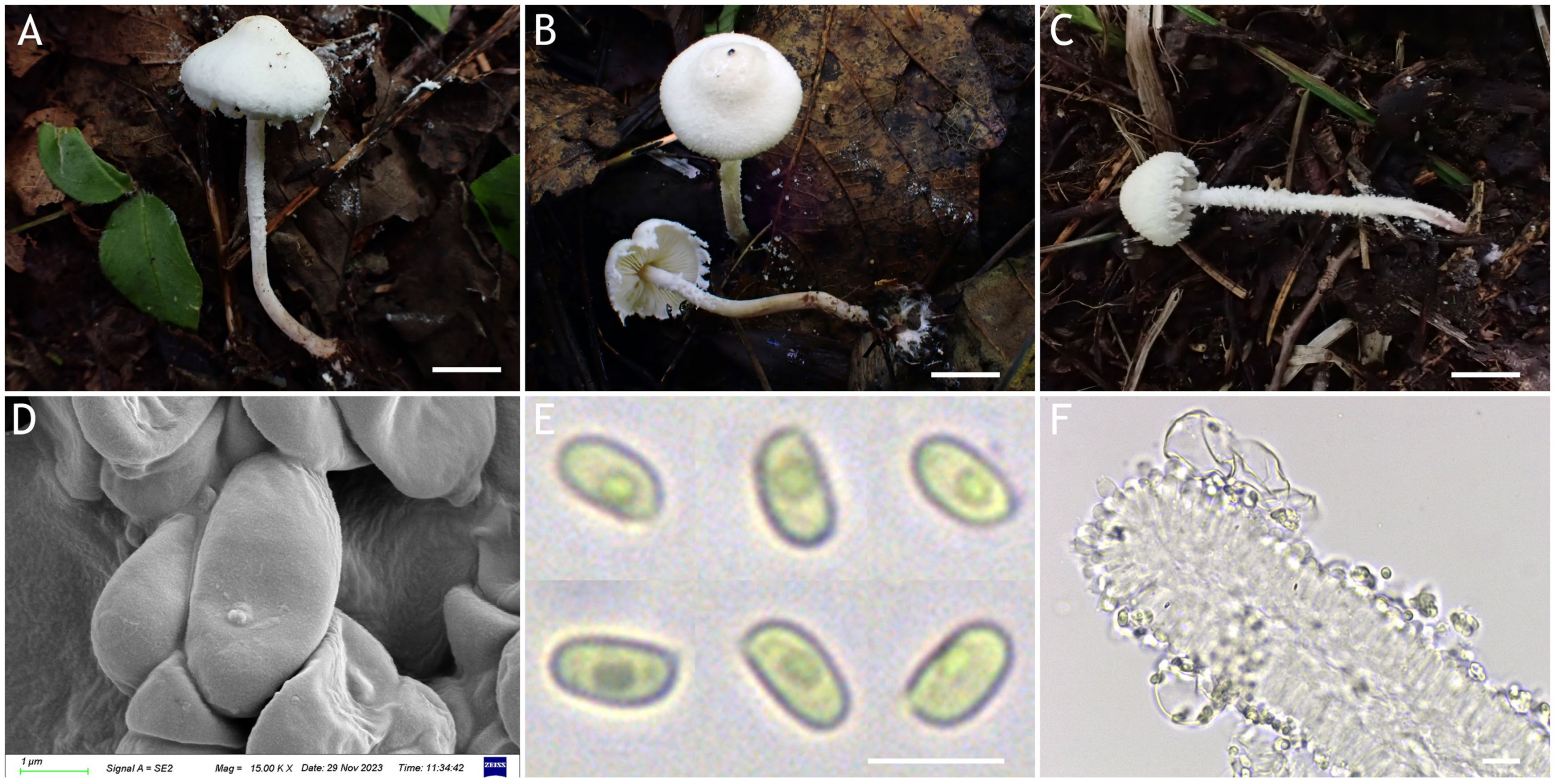
*Cystolepiota nivalis*. **(A–C)** Basidiomata, **(D)** basidiospores under SEM, **(E)** basidiospores under LM, **(F)** hyphae ends on the hymenium; **(A,D–F)** HMJAU68220 (holytype), **(B)** HMJAU68218, **(C)** HMJAU68219; bars: **A–C** = 1 cm, **E** = 5 μm, **F** = 10 μm.

**Figure 10:**
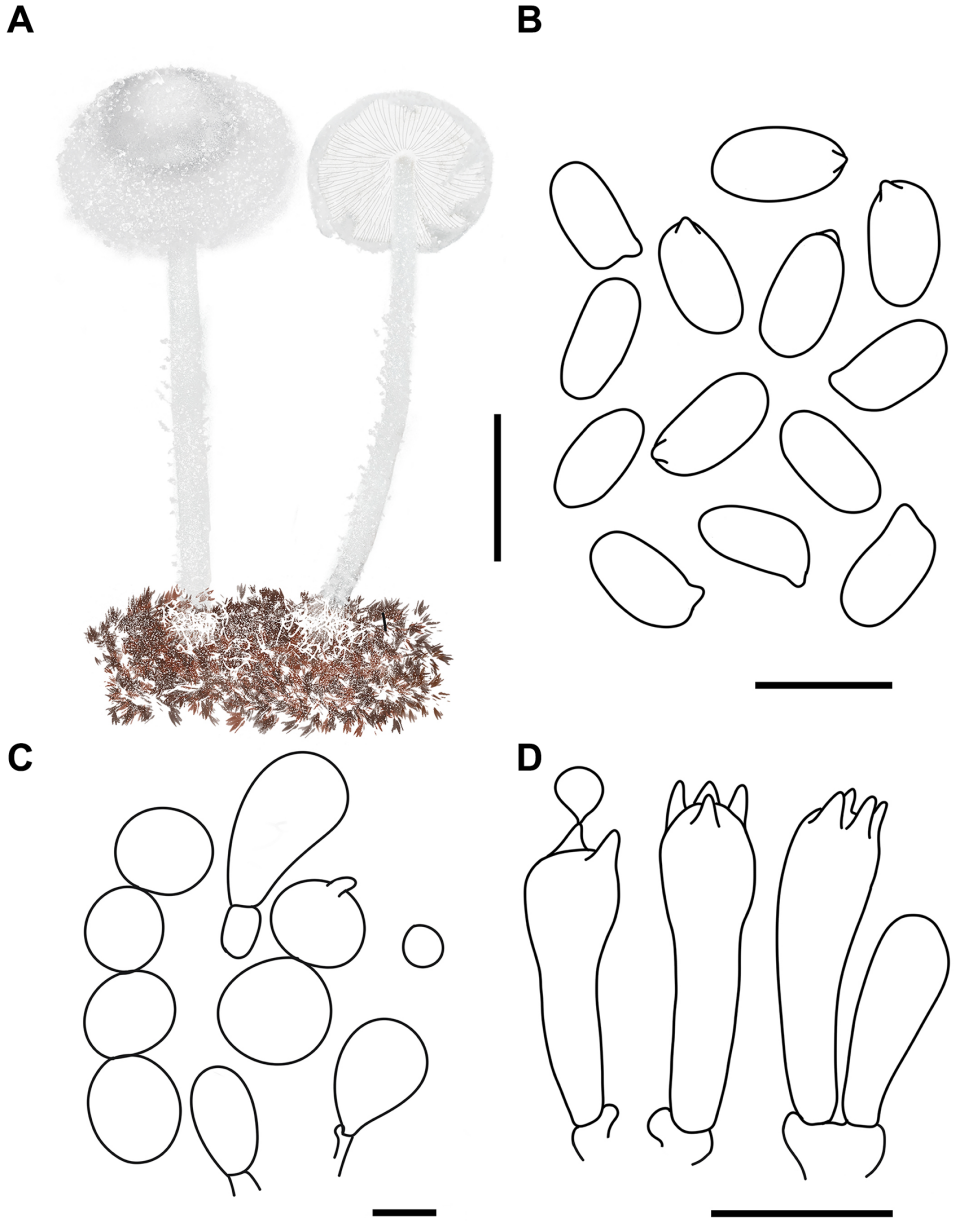
*Cystolepiota nivalis*. **(A)** Basidiomata, **(B)** basidiospores under LM, **(C)** cells of the squamules, **(D)** basidia; bars: **A** = 2 cm; **B** = 5 μm; **C** = 30 μm; **D** = 10 μm.

MycoBank number: MB 851388 (Figures 9, 10).

Diagnosis: The main distinguishing features of *C. nivalis* are the widely umbonate, white, farinose pileus, with a strongly appendiculate margin, with a farinose stipe and cystidia absent.

Etymology: “nivea” refers to snowy white pileus.

Type: China, Jilin province, Jiaohe City, Qianjin forest farm, August 25, 2023, coll. T. Bau and M. Liu (HMJAU68220), Holytype!

Description: Basidiomata small. Pileus 1.2–1.7 cm, hemispherical, then campanulate, with a broad umbo, with concolorous farinose squamules; margin appendiculate strongly farinose, concolorous with pileus; context thin, whitish. Lamellae free, crowded, white to light cream, unequal, with 1–3 tiers of lamellulae. Stipe 3.7–5.6 × 0.1–0.2 cm, central, subcylindrical to cylindrical, surface strongly farinose, white to cream, light brown (6D5–D8) to reddish brown (7E4–E8) towards the base. Annulus white, fugacious. Odorless, taste not recorded (Figures 9A–C, 10A).

Basidiospores [120,4,4] 3.8–4.7 (−4.9) × 2.0–3.0 μm, *Q* = 1.47–2.06 (−2.28), *Q*_v_ = 1.85, ellipsoid to cylindrical, slightly thick-walled, smooth-walled under the LM and SEM, hyaline inamyloid, non-dextrinoid, metachromatic in cresyl blue, cyanophilous. Basidia 12–17 × 3.5–5 (−6) μm, clavate, 4-spored, sometimes 2-spored, hyaline. Lamellar trama regular. Pleurocystidia and cheilocystidia absent. Pileus and stipe covering composed of globose, subglobose, pyriform cells 8–36 μm in diam., or 9–25 × 4–12 μm, sometimes 2–5 cells connected in a string, thin-walled, hyaline. Clamp connections present in all structures (Figures 9D–F, 10B–D).

Habitat: Solitary to scattered in mixed forest.

Distribution: Found only in Jilin Province, northwestern China.

Additional specimens examined: China, Jilin province, Jiaohe City, Qianjin forest farm, August 25, 2023, coll. T. Bau and H. Cheng (HMJAU68219); Huadian City, Red Rock National Forest Park, August 27, 2023, coll. T. Bau and X. Y. Zhou (HMJAU68217, HMJAU68218).

Notes: Morphologically, *P. petasiformis* also has a white pileus with an obvious umbonate, but it is easy to distinguish from *C. nivalis* by its context turns pale orange after cut, and lacks clamp connections (Vellinga and Huijser, 1998; Yang et al., 2019).

Key to species of *Cystolepiota* in China.

1. Pileus surface squamules fluorescent pink or greyish yellow……………………………………………………………………………2

1′. Pileus and pileus surface squamules white, cream, pale pinkish, pale yellow, light yellow brown………………………………………………………………………………………….………………………3

2. Pileus surface squamules fluorescent pink………………………………………………………………………………*C. squamulosa*

2′. Pileus surface squamules greyish yellow……………………………………………………………………………*C. luteosquamulosa*

3. Pileus and pileus surface squamules white only……………………………………..…………………………………………*C. nivalis*

3′. Pileus white to cream, pileus surface squamules white, cream, pale pinkish, pale yellow, light yellow brown……………………………………………………………………………………………………4

4. Lamellulae dry to greyish orange, light brown, greyish brown…………………………………………………………………5

4′. Lamellulae dry unchanged to greyish orange, light brown, greyish brown…………………………………………………………6

5. Pleurocystidia absent…………………….……………………….………………………………………………...*C. changbaishanensis*

5′. Pleurocystidia present, numerous…………………….……………………………………………………………………*C. fumosifolia*

6. Cheilocystidia present……………………………………………………………………………………………………………………7

6′. Cheilocystidia absent……………………………………………………………………………………………………………………9

7. Pleurocystidia absent…………………………………………………...……………………………………………………*C. adulterina*

7′. Pleurocystidia present…………………………………………………………………………………………………………………8

8. Cheilocystidia ventricose-capitate at apex, pleurocystidia rarely, occasionally clavate to fusiform……………………………… ……………………………………………………*C. pseudofumosifolia*

8′. Cheilocystidia capitate and cylindrical or moniliform excrescence at apex, pleurocystidia similar to cheilocystidia………………………………………………………………………………*C. hetieri*

9. Basidiospores strongly dextrinoid……………………………………………………………………………………*C. pseudogranulosa*

9′. Basidiospore inamyloid, non-dextrinoid………………………………………………………………………………………………10

10. Basidiospores surface rough under SEM………………………………………………….………………………………………….....11

10′. Basidiospores surface smooth under SEM………………………………………….………………………………………….12

11. Pileus expanding to plano-convex or applanate slightly umbonate, basidiospores (−3)3.5–4.5(−5) × 2–3 (−3.5) μm, *Q*_m_ = 1.55 ± 0.19, ovoid to ellipsoid……………………*C. pseudoseminuda*

11. Pileus hemispherical to convex, without umbonate, basidiospores (−3.7)4.4–5.7(−6.1) × 2–3.5 μm, *Q*_m_ = 1.89 ± 0.02, long ellipsoid…………………………………….………….*C. hongshiensis*

12. Pileus surface squamules irregular pyramidal……………………………………………………………………*C. pyramidosquamulosa*

12′. Pileus surface squamules powdery to granulose…………………………………………………………………………*C. seminuda*

## 4 Discussion

In both phylogenetic trees, the two species in *Melanophyllum* belong to *Cystolepiota*. Because a *Melanophyllum* (Velenovský, 1921) description was published earlier than that of *Cystolepiota* (Singer and Digilio, 1952), *Melanophyllum* should be used as the legal name for these two genera (Turland et al., 2018). However, the number of species in *Cystolepiota* is significantly higher than that in *Melanophyllum*. If merged, numerous synonyms can be produced. We thus applied *Cystolepiota* s.l. to both genera. We also found that no molecular data are available for many of the species in *Cystolepiota*. In particular, no molecular data is available for the model species *C. constricta*. For most species, the available molecular data is limited to ITS sequences. Other DNA regions (LSU, rpb2, tef1-α) have been sequenced for very few species. More detailed and comprehensive sampling is required to facilitate further studies of *Cystolepiota* s.l..

The macroscopic and microscopic characteristics of many *Cystolepiota* species overlap. Molecular data and phylogenetic analyses are thus necessary to identify *Cystolepiota* species with similar morphological features. For example, the species in *Cystolepiota seminuda* complex are morphologically similar. *Cystolepita hongshiensis* is a novel species examined in this study. Morphologically, *Cystolepita hongshiensis* and *C. pseudoseminuda* are similar, and require further characterization using molecular data and phylogenetic analyses. Among the *Cystolepiota seminuda* complex, we also examined *C*. aff. *seminuda* 1 and *C*. aff. *seminuda* 2. We found no morphological differences between them (Table 2). In two phylogenetic trees (Figures 1, 2), *C*. aff. *seminuda* 1 and *C*. aff. *seminuda* 2 are genetically distant from *C. seminuda*. We are thus temporarily treating it as a cryptic species.

**Table 2:** Average, minimum, and maximum of 30 mature spore measurements for *C. hongshiensis*, *C. seminuda*, *C*. aff. *seminuda*.

*C. hongshiensis*												
Specimen	HMJAU68202			HMJAU68203			HMJAU68204			HMJAU68205		
	Length	Width	*Q*	Length	Width	*Q*	Length	Width	*Q*	Length	Width	*Q*
Average	5.303	2.803	1.902	5.02	2.682	1.881	4.772	2.561	1.871	5.421	2.838	1.923
Min	4.84	2.28	1.609	4.43	2.35	1.531	3.72	1.98	1.591	4.65	2.2	1.598
Max	6.09	3.3	2.295	5.86	3.19	2.175	5.21	2.99	2.106	6.1	3.49	2.277

*C.* aff. *seminuda* 1												
Specimen	HMJAU68191			HMJAU68192			HMJAU68193			HMJAU68194		
	Length	Width	*Q*	Length	Width	*Q*	Length	Width	*Q*	Length	Width	*Q*
Average	4.384	2.349	1.876	4.042	2.271	1.788	4.116	2.25	1.84	4.043	2.233	1.822
Min	3.74	1.79	1.619	3.67	1.91	1.52	3.77	1.81	1.577	3.64	1.81	1.506
Max	4.69	2.78	2.553	4.69	2.7	2.23	4.52	2.72	2.254	4.6	2.68	2.215

*C.* aff. *seminuda* 2												
Specimen	HMJAU68196			HMJAU68197			HMJAU68198			HMJAU68199		
	Length	Width	*Q*	Length	Width	*Q*	Length	Width	*Q*	Length	Width	*Q*
Average	4.045	2.182	1.861	4.153	2.235	1.849	4.089	2.243	1.832	4.057	2.256	1.796
Min	3.46	1.86	1.591	3.81	1.86	1.57	3.7	1.93	1.548	3.62	1.79	1.576
Max	4.75	2.59	2.127	5.01	2.7	2.172	4.6	2.57	2.295	4.73	2.65	2.139
*C.* aff. *seminuda* 1 and *C.* aff. *seminud*a 2												
[240/8/8] 3.5–4.7 (−5.0) × 1.8–2.8, *Q* = 1.55–2.30, *Q*_m_ = 1.83 ± 0.03												
*C. seminuda* (Qu et al., 2023)												
[160/8/7] (−3) 3.5–4.5 (−5) × (−1.5) 2–2.5 (−3) μm, *Q* = (−1.41) 1.46–2.15 (−2.45), *Q*_m_ = 1.78 ± 0.22												

We also found that *Cystolepiota* species morphology did not correspond to phylogeny. *Cystolepiota bucknallii*, *C. rhodella*, and *C. icterina* in *Cystolepiota* sect. *Pseudoamyloideae* did not form a clade in the phylogenetic tree. They each formed a distinct long clade. *Cystolepiota luteosquamulosa* with basidiospore ornamentation did not form a clade with other species displaying basidiospore ornamentation. These require further research.

This study describes four new species belonging to *Cystolepiota* from northeast China. They are well-supported by molecular phylogenetic and morphological evidence. Thereby enriching the species diversity of *Cystolepiota* in China. In the phylogenetic trees (Figures 1, 2), *Cystolepiota* sp. (HMJAU68234, HMJAU68235, HMJAU68257), *Melanophyllum* sp. (HMJAU68255), and *Pulverolepiota* sp. (HMJAU68236) are just one specimen. The findings of this study indicate the potential existence of undiscovered species in northeast China needs to be studied further.

## Data availability statement

The datasets presented in this study can be found in online repositories. The names of the repository/repositories and accession number(s) can be found in the article/supplementary material.

## Author contributions

X-YZ: Conceptualization, Investigation, Methodology, Writing – original draft, Writing – review & editing. TB: Conceptualization, Investigation, Methodology, Resources, Writing – review & editing.

## Footnotes

^1^ https://www.ncbi.nlm.nih.gov
